# Can We Build *‘Somewhere That You Want to Go’*? Conducting Collaborative Mental Health Service Design with New Zealand’s Young People

**DOI:** 10.3390/ijerph18199983

**Published:** 2021-09-23

**Authors:** Jessica Stubbing, Kerry Gibson

**Affiliations:** School of Psychology, The University of Auckland, Auckland 1142, New Zealand; kl.gibson@auckland.ac.nz

**Keywords:** service design, mental health, youth, youth perspectives

## Abstract

Globally, young people are at high risk of mental health problems, but have poor engagement with services. Several international models have emerged seeking to address this gap by providing youth-specific care designed in collaboration with young people. In this study, 94 young people in New Zealand participated in collaborative workshops exploring their vision of an ideal mental health service. Participants were aged 16–25. Reflexive thematic analysis was used to identify seven themes. These describe the ideal mental health service for these young people as comfortable, accessible, welcoming, embedded in the community, holistic, adaptable, and youth-focused. In addition to describing how services might better serve the needs of youth, this article outlines a method for adapting international principles for youth-friendly care to the specific needs of a population of young people. This article provides supporting evidence that services should consider how to improve their engagement with youth through collaboration with local populations of young people.

## 1. Introduction

It has been well established that around three quarters of lifetime mental ill health will have onset by age 24 [1]. Given this, it is clear that youth mental health should be an important priority for not only moral and ethical reasons, but for the significant burden it places on health, families, communities, and economies across the lifespan. However, access to good quality mental health services is poor both around the world and in high income countries like New Zealand [2,3,4]. Despite having the highest incidence and prevalence of mental ill health, 12–25-year-olds have the poorest service access, highlighting the immense gap between need for and engagement with mental health services among young people [5]. This has been called a crisis in care as the majority of young people with mental health difficulties do not have their needs met [6].

### 1.1. Barriers to Help Seeking

Several factors contribute to poor mental health service access among young people. These include both internal and external barriers. External barriers are features of a service that can prevent young people from accessing care, with most research into external barriers identifying cost, hours of operation, inaccessible location, and fragmented and confusing services as key barriers to help seeking among young people [7,8,9]. Internal barriers are factors within the young person that prevent them from seeking help from formal services. These include fears about confidentiality, not feeling comfortable with services, lack of knowledge about both when to seek help and how to seek it, discomfort and shame with seeking help, and perceiving that clinicians will have negative, discriminatory, or patronising attitudes to them. [7,9,10,11,12,13,14,15,16,17,18]. Historically, most research in this area has focused on ways in which service practices can negatively impact engagement with considerably less research investigating young people’s beliefs about what services should do to reduce the barriers and improve their engagement [19].

One critical barrier to engagement is that young people are less likely to use services they perceive as irrelevant to them [18]. This is both an internal barrier, driven by beliefs among young people that services are not appropriate for them, and a structural external barrier, as traditional services are rarely designed for young people and therefore are often unsuitable for them [1,12,20,21]. At present in New Zealand, as in many other countries, youth mental health care is split between child and adolescent services for 12–17-year-olds and adult services for 18–25-year-olds, with some exceptions such as Early Psychosis Intervention Teams. Child and adolescent services are overwhelmed with long waitlists and high demand, and as such while there are many barriers to engaging in these services, these are hidden by the extreme level of need, with an additional four young people in need of services for every one who engages [1]. Despite their level of need, young people over 18 often do not meet criteria for adult services, which often focus on long-term mental health clients [22]. Both child and adolescent and adult services are often not developmentally sensitive to the needs of young people [23] and are rarely evidence-based [24,25]. This has continued to be the case despite widespread consensus that young people require different treatment approaches to either children or adults [22,26,27,28,29]. For example, it has been noted that most mental health systems rely on young people accessing services through primary health care [30] despite evidence that young people are often reluctant to engage with traditional primary health care such as general practitioners [31]. This lack of suitability to the target population could contribute to these services having poorer engagement and outcomes than any other mental health services [1,20,21], but it is difficult to determine exactly how, as these services are so rarely evaluated [25]. It is clear that the traditional model of youth mental health care is not adequately meeting the needs of young people [5].

### 1.2. Background: Youth Specific Services

Many have argued that to address this un-met need, we must respond with wide-spread, transformative change that reconsiders traditional services in favour of services that are empowering, inclusive, responsive, accessible, creative, and youth-friendly [1,5,20]. A common response to this need for change around the world has been to move towards youth-friendly services, designed specifically with young people 10–25 in mind.

The World Health Organisation have defined youth-friendly services as services that are accessible, acceptable, and appropriate to young people [32]. A number of principles have been proposed for youth-friendly services that align with this definition. These include that services should be accessible in terms of location, cost, wait time, and referral process; that they be acceptable by being safe, informal, youth-friendly, non-stigmatising, confidential, and collaborative; and that they be appropriate by offering integrated and holistic care, evidence-based practice, and early intervention [7,31,33,34,35]. It has also been proposed that services should be sustainable, through being adapted to and embedded in the community, and effectively managed [35,36].

A number of services have emerged around the world with the intention of filling this gap for youth specific care in accordance with some or all of these principles [23]. These include Australia’s headspace [37]; Jigsaw, in Ireland [5]; Maison des Adolescents, in France [38], Youth Can IMPACT and ACCESS Open Minds, in Canada [39,40]; and other programs in The Netherlands [41]; the United Kingdom [42,43]; Ireland; and Canada [23]. New Zealand currently has Youth One Stop Shops, a youth-specific integrated health care service with numerous locations. While these are not specifically mental health services, many clients would not access mental health care without the support offered through this service [44]. This service sees a high portion of indigenous Māori youth, and a significant number of their clientele have complex needs [45]. These services all tend to be conveniently located, ‘youth-friendly’, low cost, and focused on offering timely appointments with assurances of confidentiality and privacy [23].

Across youth-specific services reviewed by Hetrick and colleagues [23], 52–68% of young people saw a reduction of symptoms, suicidal ideation, and self-harm along with an improvement to function. Youth One Stop Shops in New Zealand found that 58% of those most in need of help and 52% of those with some difficulties improved after being under the service [45]. Research into headspace identified that awareness of services significantly improved in a seven-year period, suggesting that these services are able to effectively increase knowledge of services, which is a key barrier to engagement [46]. Unfortunately, due to limited research into outcomes of traditional child and adolescent mental health services, it is not currently possible to directly compare the outcomes of youth-specific services and traditional services [24,25].

However, in their review of youth specific services, Hetrick and colleagues [23] note that there are no unifying principles or standards for youth services, the details of service design are often poorly described in the research, and there is currently no single ‘best-practice’ example of youth specific services.

One component that is common to most youth specific services internationally is youth participation in the planning, design, and delivery of the mental health service [23,47]. Youth participation has been proposed as a means of improving engagement among young people by ensuring that services clearly respond to and relate to their needs and preferences [48]. Youth participatory design incorporates young people as active participants in their own care, and challenges professionals to reconsider their view of young people, shifting from disempowering and paternalistic attitudes to respecting young people’s right to agency [20]. Young people have often been positioned as passive subjects of mental health care, and attitudes to youth in mental health treatment have historically been negative—for example, beliefs that youth cannot meaningfully comment on their own care [49]. Youth participatory research builds on the notion that in order to create services that meet the needs of young people, young people must be allowed to be full and equal participants in the development process to ensure it truly responds to them [17]. In positioning young people as agents with the capacity to be drivers of transformative change, this framing draws on a long tradition of theoretical work that has pushed back against these attitudes to youth and, indeed, all peoples who have positioned as lacking knowledge and therefore power [50]. Working collaboratively with young people in this way can serve the important role of facilitating youth empowerment [51].

Many services have involved young people in the development of interventions and services [5,40,42,52] with Hetrick and colleagues [23] identifying that Jigsaw, headspace, Youth One Stop Shops, Foundry, and ACCESS Open Minds all explicitly involved young people not only in the design process, but in on-going evaluation of the service. In the headspace model, youth participation is expected at all levels including in their own care and in on-going service development, as well as high level service design, delivery, and evaluation [53]. It has been noted that this kind of involvement not only ensures that services are more relevant and appropriate to the population they are seeking to serve, but that it facilitates a youth-friendly and stigma free culture of care [54] and may be more cost-effective [55]. There is good evidence that involving young people in the process of service design is a key component to developing effective youth mental health services that address many of the identified barriers to help-seeking for young people [8,23,47,56,57,58]. It is particularly notable that some research has suggested that collaboratively designed services have the potential to engage minority young people who are traditionally underserved by conventional mental health services [59].

As such, despite the growing evidence base for some of these existing services, it would not be sufficient to simply adopt these models used in other countries. Youth participation is a key component in the success of these models, and we cannot assume that youth populations around the world are homogenous with common needs and priorities. It is therefore important to balance evidence-based service design with local adaptations [23]. It is critical for designing responsive and appropriate youth services to not recreate existing services, but to draw on the literature, methodologies, and approaches employed in service design around the world to inform the process of collaboratively developing services for specific communities and youth populations. These services have great potential to be adapted for different settings by employing a co-design process [60].

### 1.3. The New Zealand Study

In New Zealand, there is significant scope to expand the provision of youth-friendly, collaboratively designed services for young people. While there has been some acknowledgement of the importance of including service users in the design of services, this has not been specifically applied to young people [61]. The success of Youth One Stop Shops has been highlighted as an example supporting the expansion of funding for primary mental health services for young people [61]; however little attention has been paid to the role of youth engagement in their model. Youth One Stop Shops are also not explicitly mental health services, and have limited capacity to meet high and complex mental health needs [45]. As such, this research seeks to determine whether the process of collaboratively designing mental health services can be expanded upon and effectively applied with New Zealand young people to determine how mental health services might better serve their needs.

### 1.4. Research Questions

This research addresses two key research questions. These questions arose from the existing body of literature considered earlier, particularly, the development of international models of youth mental health care: [1] Do the ideals of young people in New Zealand for youth mental health services share similarities to or align with youth mental health services that have been designed internationally? [2] Do the goals of New Zealand young people for mental health services differ from the international research in any way? Do young people express why any differences are pertinent in the New Zealand cultural environment?

## 2. Materials and Methods

This study rests on a social constructionist epistemology that aims to understand people’s interpretations of their world and recognizes that these interpretations are situated within particular socio-cultural contexts [62]. Our aim in this research was to explore young people’s constructions of mental health and mental health services. Consistent with this framework, we bring together methodologies inspired by both critical empowerment research and youth agency work done in collaborative youth mental health service design around the world. A qualitative methodology was chosen to answer our research questions for its potential to elicit greater depth and breadth in the views of participants. Qualitative research aims to understand people and their interpretations of their experiences and can acknowledge that knowledge is situated within particular socio-cultural contexts, a stance that aligns well with a social constructionism epistemology [62,63].

While many methods of collaborative design have been explored in past research, there is scope to move beyond these. Traditional methods of involving young people in service design often rely on surveys, interviews, and focus groups. Each of these methods has limited capacity to elicit creative solutions and novel ideas that move beyond young people’s pre-conceptions of what services can be. For example, tendencies towards peer agreement within focus groups, lack of depth to answers on surveys, and limited perspective taking in individual interviews [64].

In this study, we applied a novel methodology intended to elicit more creative solutions to avoid participants ‘recreating the wheel’ based on what they already believe to be possible. This method was developed to overcome issues inherent in other qualitative methodologies, such as reducing the power imbalance between the researcher and the young participant [65]. This methodology enabled young people to proactively consider solutions to the problems they see, rather than focusing on their past experiences or the problems alone.

To address these limitations, we integrated traditional qualitative methodologies with participatory approaches such as those used in critical empowerment research in the broader social sciences. One such methodology is a collaborative workshop methodology, piloted by Calder-Dawe and Gavey [66] that aims to empower young people to develop solutions to problems that affect them and to improve their skills [66]. In this way, the research process is intended to not only elicit information, but also to be mutually beneficial to participants themselves in a process that is empowering to the youth who are involved. This approach was used effectively by Calder-Dawe [66] in a similar population of young New Zealanders to discuss the challenging topic of sexism. As such, it is believed a similar model could be used to safely investigate the challenging topics of mental illness and support.

We used this methodology to explore New Zealand young people’s views on creating services that serve their needs and are more likely to engage them in their time of need.

Ninety-four participants aged 16–25 with an interest or background in mental health participated in eight workshops across New Zealand. Participants responded to an advertisement calling for young people with personal experience or passion for mental health. While not all participants identified as service users (57% had been involved with mental health services), this study sought to include those who may not have accessed services due to barriers in order to reflect the perspectives of those at risk of not engaging with services.

Workshops were conducted in six different localities, including high density urban areas and townships of both the North and South Island. Table 1 describes participant demographics. The ethnic diversity of the group closely resembled the diversity of New Zealand [67] with a slight under representation of most groups and slight over representation of immigrants from the Middle East and South America and other international students. While the level of under-representation is very small, this should nonetheless be seriously considered, particularly for Māori and Pacific Islander peoples, whom are over-represented in mental ill-health statistics.

### 2.1. Data Gathering

The workshop method incorporated individual surveys at the beginning and ending of each workshop to elicit initial thoughts and any responses that might not be expressed, such as if they would be considered socially undesirable. A group discussion more typical of a focus group then occurred, followed by an activity in which young people responded to a prompt—a newspaper article about a proposed new mental health service. Lastly, participants engaged in a creative group project in which they worked in teams to design an ideal mental health service, which they then presented to the full group. It was noted that, in addition to addressing some challenges of qualitative research and eliciting nuanced and creative ideas, the mixed methods utilised in the workshops also allowed different participants to ‘shine’ in different activities, with some very vocal in discussions, others writing more, some annotating their newspaper articles, and others drawing services. Each workshop lasted between two and three hours dependent on group size and were facilitated by the lead author, a training psychologist, and doctoral candidate. The size of the focus group varied from three to twenty-two participants.

### 2.2. Data Analysis

The data was analysed using Braun and Clarke’s [68,69] framework for reflexive thematic analysis, which involves identifying themes that reflect important trends in the data relevant to the research question. The approach employed in this thematic analysis was inductive, focused upon identifying themes within the words of participants. It is important to note that many alternative approaches to thematic analysis and qualitative research more broadly are employed by different researchers. A reflexive thematic analysis approach was chosen for its appropriateness for our social constructionist epistemology and its value in highlighting the distinctive ways that youth in the current New Zealand context might view mental health and mental health services.

Data were first transcribed and combined with written survey answers. Prior to analysis, all identifying information was removed from the transcripts. Due to the nature of the data collected, it was not possible to identify individual participants’ contributions consistently throughout the transcripts or anonymized surveys, and as such, participant numbers could not be appropriately assigned.

The analysis began with immersion in the data and the full transcripts were read several times. All statements that related to young people’s attitudes to or ideals for mental health services were then extracted. These statements were then tentatively grouped into overarching categories that related to similar subjects using NVivo software for support given the large amount of data. Table 2 reflects the categories that these statements were grouped into. The purpose of these categories was to identify the richness and diversity of content and aide the process of theme formation. Categories were fluid and organic rather than prescriptive, as is recommended in reflexive thematic analysis [69]. These were then refined and shaped into themes. The process of refining themes from the transcripts is not a linear one, and this process was iterated several times and reviewed by both researchers until it was felt they accurately reflected the data. To ensure trustworthiness of the analysis, the themes were discussed, reviewed, and refined by both researchers at each stage of the analysis to ensure consensus and increase fidelity [70]. This practice ensures thematic analysis is a true reflection of the data and avoids any individual author’s perspectives overshadowing the opinions of participants [70].

Examples that illustrated the beliefs of participants within each theme were extracted from the transcripts. Any remaining identifying information included in the examples was removed. Descriptors such as ‘many’ and ‘a few’ were used to indicate how frequently themes or specific ideas within a theme were addressed by participants, but are not intended to imply the possibility of statistical measurement.

Establishing rigor is important in qualitative research. Reflexivity is regarded as an important criterion for establishing the quality of qualitative research, particularly reflexive thematic analysis [70,71]. Reflexive thematic analysis practice acknowledges that meaning making is inherently contextual and that it is therefore important for authors to be aware of their perspectives and the influence of these upon the research, rather than seek to ‘eliminate’ the presence of the author from the project [70]. For example, the very selection of a research topic is a reflection of the researcher’s backgrounds and interests. As such, we wish to acknowledge our positioning and how we sought to balance these with rigorous, high-quality reflexive thematic analysis. The first author is of Māori and European heritage. She is a student on a doctoral clinical psychology program and a young person who fits in the age range of our participants. The second author, a South African migrant to New Zealand, is a researcher and psychologist with experience of working clinically with young people. Given our professional positioning, it was important to resist and challenge tendencies to confine the accounts of participants into dominant scientific explanations or notions of mental health services, and to pay attention to the different and novel ideas young people were proposing. Similarly, given our cultural backgrounds it was important to focus on participants’ words rather than making assumptions about what this might mean within specific cultural contexts. In addition to reflexivity, Morrow [71] has identified a number of other important ways of establishing the rigor of research conducted from a social constructionist position. Dependability is assured by providing a detailed account of the procedures we undertook through the research in both the data collection and analysis. To facilitate this, written records in a digital journal were kept at each stage of the data analysis process, including records of meetings and subsequent changes to the themes throughout the analytic process. Rigor also calls for a process of collaborative meaning making. The methodology in this study allowed for ongoing exchange of meaning between the researchers and the participants during the process of data collection. This collaborative meaning making extended to the co-researchers involved in the project and analysis involved close consultation with one another and reflection upon our perspectives on the issues present within the transcripts including how our views differed and aligned with those of participants. This occurred within regular supervision sessions and discussions of the data analysis process, including referencing and re-reading the complete transcripts throughout analysis.

### 2.3. Ethical Considerations

Ethics approval was granted by the University of Auckland Human Participants Ethics Committee. The researchers recognised the sensitivity of the research and care was taken to establish the safety of participants. Workshop conversations were monitored for content suggesting distress, and procedures were in place to support young people who were considered in need of services or intervention on the basis of conversation. Participants were also provided with contact information for support services should they need it.

## 3. Results

Seven themes were identified through the thematic analysis that reflect the kind of mental health service young people want. This is a place that is comfortable, accessible, welcoming, embedded in the community, holistic, adaptable, and youth focussed. It is the combination of these themes that most accurately reflects the wishes of the young people who participated in our workshops, none of these themes in isolation would be sufficient for designing a youth friendly mental health service.

### 3.1. A Place That Is Comfortable

Across our workshops, young people described the kind of mental health service they would like to attend as a comfortable space in which they could feel safe and relaxed. As one young person put it, they sought a space “where [young people] feel like they can be honest and at ease and not like they’re in an asylum”.

Participants spoke to aspects of design that could increase comfort. For several, this meant reconsidering the clinical space, which had often been experienced as uncomfortable due to features like dim lighting, plain design, or classical music. As this young person said: “It was just like, kind of a dark room with weird ambience like a weird and not like bright and happy”.

Instead, young people described the ideal service as more relaxed and informal spaces with brighter colours and lighting, and more unconventional furnishings including giant bouncy balls, comfy couches, and bean bags. Many young people spoke about the balance between making a space fun and engaging for them without being too childish or feeling too young. This young person described it like this:

*I don’t want to say like make it look like a kindergarten, but you know like make it a really happy place to be with lots and colours and stuff. Because a lot of the places you go they literally look like a hospital room or like a business office and you always think of the scenes in like movies where they’re lying down on the couch with the clipboard and that’s really not inviting*.

Several young people’s ideal service offered options outside of the traditional ‘two chairs and door’ model, where they could do activities while speaking with their clinician. Examples including playing games, weighted blankets, or playing with stress balls or fidget spinners. This young person described how different approaches might work at different times:

*Some days you don’t want to talk about your feelings, but you want someone to just chill out with. And sometimes you do want to talk about your feelings! So... like a chill room and a professional formal room. So, the Chill Zone... if you just want to come in after school and you’ve had a bad day and you just want to watch some Netflix with your counsellor, maybe you want to play some board games, maybe you want to play with the stress ball while you’re talking or you know listen to some chill beats. And there’s some bean bags. And then this is the formal zone, with the chair and a couch and a glass of water because it’s formal, there’s not too much going on and there’s not many distractions. But it’s a place where you can get down to business because sometimes that’s what you want*.

Many young people wanted a space to be more reflective of youth culture. For example, a space playing popular music, having posters for upcoming events, or having Wi-Fi: “For young people, it would be like kind of reflective of like pop culture or things that they can kind of relate to. So, like, nice colours and like photos and posters and stuff for upcoming events”.

For other young people, their desire was for a relaxed and soothing service with an attractive and calming appearance, often including plants:

*I was thinking like a tranquil environment. So, to me that would be like water fountain thingies and like really calm like nice music. So, like, something that’s really just relaxing so you can just really exist in the moment*.

Many young people spoke about how they would want to feel welcomed into this space from the time of arriving in the reception. One group described this feeling of welcoming as treating you as if you are house guests, such as by offering food and drinks.

Finally, young people spoke about hoping to see more of their clinician’s personalities within the spaces they work, forgoing bland furnishings and generic offices for personal spaces that could show them more about the person they were working with. One group member, who had experienced this in a private service, described it like this:

*Like in some private clinics, the doctor’s office is a bit of a reflection of themselves. But in public service, you’re in a very bland room which can kind of make you feel like you’re in an asylum. Which is not an atmosphere you want to be feeling ever. It also gives you a sense of the personality of the person you’re dealing with, like you’re dealing with a person, you’re not dealing with a name, a title*.

### 3.2. A Place That Is Accessible

Young people who participated in our workshops consistently expressed their desire for services that are more accessible to them. For many, location was a key issue in accessibility, with many wanting more local services. This group from a large city spoke about the need for services around the city rather than in one central area where many might not access them: “It would be within easy driving distance to people. Maybe one in each like major area. Like on in south, west, central, east. Like the most densely populated areas or around where students are”.

Other young people emphasised the importance of these locations being spaces regularly frequented by young people, like malls, similar to the model for Family Planning in New Zealand. They spoke about how this can be less awkward for them:

*It needs to be somewhere a lot of teenagers go, so it’s like natural for them to go there*.

*Like at a mall. Like family planning or some stuff*.

Other participants, particularly in smaller towns, proposed novel solutions to the difficulty of more distant services. Many proposed services on popular public transport lines, such as trains or popular bus routes. Others proposed shuttles that young people could book spaces on. However, several proposed mobile clinics that could attend their schools or visit their towns. These were often based on the popular dental nurse vans that visit New Zealand primary schools to conduct annual dental appointments free of charge to students:

*We talked about mobile vans. So, like you know how we have like the dental van that comes to school? So, like having maybe something similar so then more people, like it’s less of an intense approach than going to talk to the counsellors...so people coming to your school like somewhat regularly, like you do with the dental van, they come like every few months*.

In addition to being physically accessible, young people spoke of the need for services with accessible cost. Almost all young people expressed a desire for free services for youth, with some suggesting a very cheap service or a sliding scale based on personal income rather than parental income. This group decided:

*Free through all school and then cheaper for like five years after uni. Because they might still be experiencing stress, you know, getting their life sorted trying to find an actual job. But free services available to like, school attendees and universities*.

### 3.3. A Place That Will Welcome Me

Young people across our workshops described their ideal service as a place that they will be welcomed to if they make contact. Many compared this to services where they had reached out for help only to be turned away.

For many, this meant decreasing the wait times for services, most often to a week. Several participants spoke about how long delays between making contact and receiving appointments are harmful to young people who may wait until their challenges are acute to even reach out. As this participant said:

*I want it like, to be able to actually talk to someone one on one when you need to [should be] much easier, because I know one of my friends that like literally had to wait for like six months before she got a place to talk to someone and by that time it was like... got a bit too far*.

Several participants spoke about how a service that was easy to get in contact with would be perceived as more open to them. For many participants, this was as simple as having email addresses available or operating a phone line including emergency helplines. Other participants had more novel ideas for how to get in touch with the service, including via social media:

*I think social media. And then there could be someone managing the reception desk that just gets in the messages from social media and like a computer system that filters through it because I imagine there could be quite a lot*.

Several groups proposed using apps to make contact with services or for triaging:

*Having like a free app that people can get... like you could go onto this app and just discuss how you’re gonna feel and then the people that run like the reception can have a look and try to get you to talk a little bit more about what you kind of need and then they’d like guide you to where you need to be [within the service]*.

Many young people spoke about changing the approach to referrals to not rely on professional referrals: “Maybe a way of going so they can feel like they don’t have to see their GP if they’re not providing the right support or recommendations for them”.

Several participants spoke about peer referrals as a way into services for young people who are struggling to ask for help themselves. This participant described an idea where young people could contact a service about a friend they were worried about:

*Like, if you’re not in a position to engage directly, someone you know might be able to come to them with a concern and they can get in contact with you so that they can outline what support they can offer*.

Others spoke about how a welcoming service would be one with which the initial contact was not intimidating or stressful, such as when meeting with psychiatrists as first point of contact. Young people spoke about the ideal service as a space where they did not feel stigmatised for asking for help. This young person phrased it as: “Something more like, you can approach them without it feeling like you’ve got something wrong with you”.

### 3.4. A Place That Is Embedded in the Community

In our workshops, many young people spoke about their ideal mental health service as embedded and visible within their communities, rather than a siloed space for therapy only. For many, this included increasing visibility and awareness of services:

*I think the big part is raising the awareness of it. Because I know lots of people might not know about the services or they might not think they’re eligible or like at the level that needs to go talk to someone about what’s going on*.

Ideas for how this could be done included advertisements using posters, television shows, social media, and painting billboards or buildings. As this young person put it:

*It needs to be easily visible, like advertising. Cause if I’m walking around and then like I see a poster about like mental health or whatever and it has like a phone number, an address, an email, or whatever, then I can just you know email them, call them, go there, get help. But if there’s nothing to advertise it, then I don’t know where the hell I’m gonna go and then it just becomes like, well no one can get help because they don’t know where to get help*.

Several young people spoke about how this advertising should be done with the input of young people to ensure it appeals to them as potential service users. This young person said:

*If there was like a young person who was in charge of advertising on social media, rather than a 30-year-old being like ‘hey, kids, you wanna nae nae on down to the counselling office and we’ll help you out.’* 

Many participants extended this beyond simple visibility to active presence within the community, such as by speaking about the service at local schools or through presentations to the broader community. Others spoke about how services could hold events for the broader youth community in an area, rather than exclusively providing services for mental health treatment. This group proposed this:

*I think it would be really cool to see like events where it was a combination of like, if we did young people it could be open to anyone. People struggling with mental health and then people who are not to create that awareness. Just like, events open to the public*.

Some young people spoke about a desire that these services could be involved in training other professionals who interact with them, including their teachers, parents, and health professionals, to help them know how to talk about mental health and what help to offer:

*It doesn’t have to train the teachers to be really good to provide counselling, but maybe to spot like ‘oh, maybe this student’s not doing really well’ and then maybe reach out and see if the student needs help. If we educated teachers on mental health then they’ll be more aware of it and they can actually play a part, so you can actually help them in a school environment. And if the students are outside then it could be family, friends, professionals... so it’s like the inside out everywhere kind of thing*.

Lastly, young people also spoke about how services could be involved in providing information that is readily available to young people about mental health and services, particularly information that can be found online. As this young person said:

*Even if you google stuff it doesn’t really come up with everything. And then you have to google another keyword and it’s like, I don’t know. Like everything about counselling feels so outdated, they need to update everything... Like, easier to navigate through them. I’ve tried and it’s just like all this tiny ass print all together and there’s just this ugly photo in like this ugly green background and it’s really overwhelming*.

### 3.5. A Place That Treats Us Holistically

Many participants in our workshops described their ideal mental health services as holistic, considering multiple aspects of a person’s mental health including physical health, social health, and spiritual health: “I think mental health has got a few aspects which is like physical, mental, social, and I think one is spiritual. So, I think it’s more the integration that would actually help”.

Several participants particularly emphasised their desire for a space within the service to engage in activities to support their physical health, such as by having a gym within a service. Others proposed having healthy cooking classes, particularly for young people moving out. This young person said:

*I guess for me, I’m someone that prefers like really practical stuff. Like I love exercise and I feel like that’s a huge part of mental health and staying well, so that’s something I would like to see in the mental health services*.

Other participants spoke about the importance of a mental health service that also promotes social wellbeing, particularly through providing recreational spaces for young people to interact with one another. This group emphasised peer support as a way to serve this purpose: “You could just like, have someone creating a space where young people going through mental health can come together and support each other, and just like hash it out”.

Many participants sought services that provided more opportunities for spiritual wellbeing, however this looked to them. Some young people suggested having workshops for traditional healing practices that service users could participate in. For many participants, this was as simple as having clinicians from different faith backgrounds available:

*I wonder if we can include like a chaplain or some kind of person with a faith background. Because I feel like sometimes with mental health services they don’t really acknowledge people’s faith in their system. Especially because like, some churches people go to might also have a stigma around*.

For others, spiritual health meant access to spaces where they could feel at peace and a sense of calm. This often meant yoga rooms or garden spaces. As this participant said:

*We wanted to include a rooftop garden because some people feel quite closed in when they’re in a room, so if they were able to have a bit of a good view and just fresh air then they can feel more at peace to talk about issues*.

Some young people’s ideal mental health service also incorporated online options, discussing how this could be helpful given the amount of time young people spend online: “I can see the counselling services online and you can just chat with a counsellor sometime. And I think it’s really helpful”.

### 3.6. A Place That Is Adaptable

Many young people in our workshops’ ideal mental health service would be able to adapt to their individual needs. These included options for group and individual therapy, or to bring friends to sessions. Many of these young people found talking therapies awkward and unhelpful for their specific needs:

*Mental health is very different to any other kind of service that could be like provided to us. Because say like at a hospital you can just be prescribed to the same drug as everybody else and you probably have the same results. But if you’re in mental health and you just have the same thing told to you, like it’ll probably be far less effective because it’s all individual and it just doesn’t work to like fit everyone into the same kind of box*.

Many suggested offering different treatment approaches that could be designed to serve their individual needs:

*In the end, you’d have an outline of at least two different strategies so you don’t feel boxed into one thing. And if they suggest a medication, they should always have another option.... Whether it be cognitive behaviour therapy, dialectic behaviour therapy, occupational therapy, they can suggest these things as well, but not saying you’re going to have to do all this. Just, this is what I recommend as a starting point and your treatment plan will be refined along the way*.

Many participants described an ideal service as offering diverse clinicians, including occupational therapists, speech language therapists, counsellors, nurses, psychologists, and psychiatrists. Participants hoped that these clinicians would offer different models of work beyond traditional counselling:

*I would prefer to see them putting money into occupational therapists and training them up to help people. Because I know that with the team of specialists that I worked with, the person that was most helpful for me was my OT, because she did like really practical things with me*.

Other young people spoke how options could include specific therapy models like Mindfulness, Cognitive Behaviour Therapy, and Dialectic Behaviour Therapy to manage significant mental health problems and trauma. Some of these young people spoke about how the assistance they had been offered was not sufficient to fully address their needs:

*Cognitive behavioural therapy, like I’ve heard of it. I would’ve liked to have that available. Cause yeah [they] were like ‘you should do cognitive behaviour therapy’ and it’s like, where am I going to go to do it? Because they can’t do it*.

One group proposed an approach to an adaptable service that could involve young people being involved in tailoring their own treatment plan. Under this model, young people could sign up for a range of programs recommended by their clinical team: “There could be like a rotation of programs. Like you go inside the app and you find that there’s like set events or like counselling methods that you could sign yourself up to for a certain amount of time”.

Some participants spoke about desiring more specialised clinicians with specific skills that could be tailored to the areas of concern for them. Other participants disagreed with this, emphasising the high chance of multiple issues. However, others described feeling that their clinicians were not specialised to understand what they really needed. This young person described it like this:

*It should be different counsellors that study different like departments. So, it’s more personalised to go to not just one counsellor that knows a bit of everything, if you’ve got a specific problem you’ve got a specific person that can help you*.

Young people reported that their ideal mental health service would be flexible with them about the degree to which they would like their family involved. This participant expressed:

*There’ll be some people out there that don’t want their parents involved, or they might want to be there when they’re talking to their parents, or they might not want to be. Or they might want their parents to only know certain things. And they can ask you ‘are you comfortable with us sharing everything or are there certain things you want us to leave out?’* 

Additionally, young people who participated in our workshops spoke to the need for services that can adapt to be more culturally responsive, by being respectful of the mental health needs and challenges of different cultural groups. This participant put it simply: “Like, the services should be culturally sensitive”.

As our workshops took place in New Zealand, many participants, both Māori and non-Māori, emphasised the need for services that are understanding of Māori cultural needs. As this participant said:

*I heard that not many services are being provided for Māori.... It’d be really good if they had services provided to them, that it’s open to them, and since they have cultural differences so... it’s part of the therapist’s plan to make that service open for them, make it a bit more approachable, and follow their roots, follow their traditions and customs*.

Others stated that all cultures present in New Zealand should be included and have responsive services. These participants made reference to how different attitudes to mental health may make it challenging for immigrants, with specific mention of young people from the Pacific, Asia, the Middle East, and Africa. As this young person said: “I think all the cultures that are residing in New Zealand should be included because they would have different ideas for how a psychologist should be. Especially now that New Zealand is becoming more diverse”.

### 3.7. A Place That Is Youth Focussed

The young people who participated in our workshops emphasised their desire for services that are youth focussed and tailored to the needs of young people. The specific age limit proposed ranged from a minimum between 11 and 16, with the age limit either at 20 or at 25. The most common age grouping was 13–25. Specifically, many participants spoke about the need for youth specific services that were distinct from child services. This participant stated: “You obviously can’t treat a five-year-old the same way you would treat a 16-year-old”.

Participants gave examples of being talked to like a baby, and how they would prefer a service where they were treated like an equal. Similarly, they emphasised that this service would not treat young people like adults: “You have like a targeted youth program specifically for young people, because let’s say it’s open to the adults for example, then you will have more specific targets. Obviously adults have stress of like, adult issues”.

Other aspects that young people emphasised in their ideal service was that it would be a transparent and honest space that puts you at a more even playing field, rather than feeling that there is a significant power imbalance between the young person and the clinician.

Some participants emphasised how a youth responsive service would not stigmatise young people or suggest that they should not be struggling because of their age, or assume their problems are age related and will pass. This young person stated an ideal service should be: “Somewhere they can seek help without feeling judged, like ‘you’re young, why do you have all this stress’”.

Finally, participants noted that services should be responsive to the perspectives of young people, such as with suggestions boxes for young people to report what they would like from the service.

## 4. Discussion

This study identified seven themes that describe the ideal mental health service for the young people who participated in our research. These themes were a place that is comfortable, accessible, welcoming, embedded in the community, holistic, adaptable, and youth focussed. These findings are consistent with a large body of international research into youth preferences for mental health services, and additionally offers a guideline for how these principles could be applied to services in the New Zealand context. This research also offers a methodology that could be used in other cultural contexts to adapt best practice principles for services to fit the needs of a local population.

The first research question proposed at the outset of this study was concerned with whether the perspectives of young people in New Zealand would align with international research regarding youth friendly mental health care. A number of general principles for successful youth mental health services have been defined in the research. These include that services be youth-centred and holistic; accessible in location, hours, referral process, timeliness, and cost; that they be informal non-stigmatising environments; provide recreational or drop in space; that they be integrated into the community; confidential; evidence based; staffed by welcoming and youth focused clinicians, and that young people should participate in the planning, delivery and evaluation of services [7,31,33,34,35]. These principles align very closely with the ideal mental health service proposed by young people in our study, with all of these emerging in some way across these themes. This provides substantial supporting evidence for these principles, identified here in an independent sample of young people not explicitly exposed to these ideas prior to participating in this research.

While these international principles for youth-specific services have been proposed, researchers have highlighted the importance of balancing international standards with local adaptations, to ensure services are relevant to their specific target populations [23]. Importantly, our findings also share a number of similarities to ideas that were highlighted in a 2020 review of stakeholder feedback on child and adolescent mental health care in a New Zealand district health board [35]. These consistencies included that stakeholders believed treatment outcomes for young people would improve with more active involvement of family and clients, individually tailoring treatment plans, and including multi-disciplinary staff trained in treatment to offer multiple models of care. They also emphasised accessibility through affordability, convenience, and timeliness; acceptability through being youth-friendly, confidential, and respectful; appropriate care, through a developmentally appropriate and evidence-based focus; and sustainability, through being embedded in the community. These results again share a high degree of theoretical similarity with the results of this study, which is particularly significant for how this demonstrates congruence between the ideals of other stakeholders and those of young people. This demonstrates that our findings are not only consistent with international practice standards and principles for youth-friendly services, but with the beliefs of an independent sample of local youth mental health stakeholders.

Our second question was concerned with any key differences between the perspectives of young people in our study and the international research, and where such differences might arise from. In their 2017 paper, Hughes and colleagues [51] proposed a set of ten principles for best practice in youth mental health services. Consistent with Hughes and colleagues’ principles, our participants emphasised the importance of family needs, holistic care, empowering and developmentally appropriate services with a youth-friendly approach, easy access, and evidence-based practice. However, young people in our study did not discuss prioritizing high-risk youth or covering the spectrum of care in one service. This may highlight that for this sample of young people, many felt their needs had been neglected due to not being ‘severe’ enough and as such most emphasised the importance of early intervention over care for the most ‘severely’ unwell. Another explanation may be that some young people did not have experience with the full spectrum of severity.

It has been noted that models for youth-specific services that are emerging around the world have the potential to be adapted for different local contexts through the process of co-design [60]. The value of local co-design may lie in allowing researchers to specifically tailor the application of these principles to the needs of the local community. This also addresses the challenge identified in Hetrick and colleagues 2017 review [23] that research often poorly describes what these principles look like when applied in practice to the design of a youth-specific service. For example, what does a youth-friendly service that is comfortably and attractively designed actually look like? While one service might be designed in an appropriate ‘youth-friendly’ style for their setting, this might not be considered a comfortable environment to all young people around the world. While these principles might clearly illustrate a guiding framework for services, they do not often offer the detail and practical examples of how these can be applied in practice to specific communities of young people.

Young people in this study did identify a number of clear and practical ways that a service could be designed that also aligns with these principles. For example, in order to be accessible, services should consider having a presence on social media and options for self-referral online or through apps. To be comfortable, services should offer relaxing spaces playing popular music, with games and activities to enjoy like pool tables and video games. To be holistic, services should include a range of therapeutic modalities delivered by a multi- disciplinary team, and should include spaces to exercise, learn practical skills like cooking, and complete both individual and group therapy. For our participants, ‘youth-focused’ care included care delivered by clinicians specialised in work with young people who provide treatments adapted for their developmental stage, rather than treatments designed for younger children or adults. This finding is particularly significant as ‘youth- friendliness’ is generally poorly defined in literature on youth-specific services, with even the consistent practices across services [e.g., ‘bright and comfortable’] providing little guidance to those seeking to design services as to how a service can practically become more ‘youth-friendly’ [72].

The results of this study suggest that while principles of youth-friendly services may be consistent internationally, the detailed guidance of how to apply these principles in a specific local context should come directly from the community. Young people were easily able to express their desires in a clear, practical way that could be applied to the design of a service. As such, rather than attempting to consult a body of research for detail that is not present on specific design qualities, service designers would better spend their time conducting their own co-design workshops to elicit the specific practical ideas of young people in their community. The design process should therefore be rooted in adapting international best-practice principles to a practical and specific design that is developed in collaboration with the local community. This workshop method is one proposed methodology by which this could be done, which worked effectively with our sample to elicit detailed and helpful responses.

This approach is consistent with the core common value of all youth-specific services, which is to include the voices of young people [23,47,72]. To simply reproduce an internationally designed service in a new context with different young people would directly oppose this value by neglecting the specific voices, goals, and needs of local youth. Indeed, it is likely that services are more successful when well-adapted to the specific needs of the local community [36]. Youth culture is rapidly changing and flexible, and can look very different around the world. As such, there can be no single image of what ‘youth-friendly’ would look like in all contexts and there is likely to be a lot of variation in young people’s preferences [73]. Above all else, services should continue to prioritise the preferences of the young people accessing the care they provide and should remain adaptable as their desires and needs adjust.

### 4.1. Limitations

As noted in this paper, in order to be truly youth-focussed services must include the voices of their target population in their design process. All young people who participated in this project volunteered, and this opportunistic sampling is not necessarily reflective of the perspectives of the broader youth population. Our sample is likely to represent young people who are interested in mental health and who have knowledge and awareness of this, and may not reflect the concerns and experiences of those without this knowledge. This sample was diverse and reflective of many groups from around New Zealand, but was slightly under representative of some key demographics, including Māori and Pasifika young people. Our sample is also under-representative of men, which is unfortunately common in some mental health research [74]. This could relate to limited help-seeking among men and reminds us of the importance of male-focussed psychological research [75]. Given the low number of male participants, we were not able to conduct any analysis comparing the perspectives of young people of different genders. Additionally, our representation of gender non-conforming young people is low. Given the particular vulnerabilities of this group, it is important that research considering their specific needs is conducted. Finally, it was beyond the scope of this study to consider the needs of young people engaged in juvenile justice or whom have disengaged from formal education. These young people are very likely to have alternative perspectives on how services could best meet their needs. As such, further research exploring the perspectives of these groups would be important to designing a service that is truly reflective of the needs of New Zealand’s young people.

### 4.2. Implications

This study provides further evidence that young people are developmentally capable of taking agency over their mental health care, and can lead nuanced and informative discussions about the design of services intended for their use.

When reviewing the priorities and ideals discussed by the participants in this study, there appears to be a significant gap between the current youth mental health system and what young people are hoping to experience in their care. Generally, participants desired a stable service specifically designed to provide care for pre-teens, adolescents, and young adults. In much of the world, it may not be feasible or realistic for services to entirely overhaul their care for young people in favour of dedicated specialist services. This paper provides evidence that all services who seek to improve their youth-friendliness are likely to benefit from collaborative input from young people within their target population. Importantly, the present study suggests that approaches to mental health care such as increasing funding for traditional primary care may not be effective with young people, who are less likely to engage with traditional primary care [31] and whose needs are not met by these services. Services that seek to improve the youth-friendliness of their practice should do so through collaborative consultation with youth and participatory consideration of the research base, rather than assumptions about how to improve mental health care made without the input of young people.

Finally, the results of the present study indicate that clinicians are critical to the experience of services for young people. Mental health services must prioritise the training and retention of youth-friendly clinical staff to maximize their youth-friendliness and efficacy.

## 5. Conclusions

Around the world, youth-specific mental health services are emerging as a potential solution to the problem of low engagement of youth in mental health services, despite high need. A number of international principles have been proposed based on these existing models, which describe the qualities of a youth-focused mental health service. In this study, we utilized a novel methodology to explore the preferences of young people in New Zealand regarding mental health service design. We identified a high degree of similarity between the perspectives of our independent sample and results found internationally. We also demonstrated that when given opportunities to explore creative ideas, young people are able to give detailed and nuanced descriptions of specific and practical ways in which services can become more youth friendly. As such, all services for young people should involve youth voices in their design process as they are clearly capable of being involved in this way. The methodology laid out in this paper may be one effective way of hearing and incorporating youth perspectives.

## Figures and Tables

**Table 1:** Participant Demographics.

Variable	Percent of Sample
Gender	
Male	19%
Female	80%
Non-Binary	1%
Ethnicity	
New Zealand European/Pakeha	68%
Māori, Indigenous New Zealanders	15%
Asian (Mixed Origins)	10%
Pacific Islanders	6%
Other (Middle Eastern, South American, European and Asian international students)	9%
Sexuality	
LGBTQIA+ *	16%
Variable	
Age	
Range	16–25
Mean	17.77

* Individuals who self-identified as Lesbian, Gay, Bisexual, Transgender, Queer/Questioning, Intersex, Asexual, or any other sexual identity other than heterosexual/‘straight’.

**Table 2:** Initial categorization codes.

Topic	Categories
Services	Specific Services
	- Physical Clinic
	- Mobile Clinics
	- Youth Programs
	- Training for Laypeople
	- Adapting Existing Services
	* School Counselling*
	Service Environment
	- Appearance
	- Location
	- Facilities
	Logistics
	- Target Populations
	- Referrals
	- Cost
	- Age Range
	- Duration
	- Wait Times
	- Contact
	- Role of Friends and Family
	- Use of Technology
Awareness	Session Structure
	Models
	- Early Intervention or Prevention
	- Medication
	- Evidence Based Therapy
	- Talking Therapy
	- Alternative Therapies
	- Social Work
	- Cross-Cultural Approaches
	- Choice
	Culture
	- Confidential
	- Transparent
	- Culturally Sensitive
	- Tailored
	- Respectful
	* Judgment Free*
	- Reducing Stigma
	- Rethinking Approach to Suicide
	- Agency
	* Empowering Collaborative*
	- Choice
	- Approachable
